# Impact of Reduced Cerebellar EAAT Expression on Purkinje Cell Firing Pattern of NPC1-deficient Mice

**DOI:** 10.1038/s41598-018-21805-z

**Published:** 2018-02-20

**Authors:** Michael Rabenstein, Franziska Peter, Arndt Rolfs, Moritz J. Frech

**Affiliations:** Albrecht-Kossel-Institute for Neuroregeneration (AKos), University Medicine Rostock, Gehlsheimer Straße 20, D-18147 Rostock, Germany

## Abstract

Niemann-Pick disease Type C1 (NPC1) is a rare hereditary neurodegenerative disease. NPC1-patients suffer, amongst others, from ataxia, based on a loss of cerebellar Purkinje cells (PCs). Impaired expression/function of excitatory amino acid transporters (EAATs) are suspected of contributing to PC-degeneration in hereditary spinocerebellar ataxias (SCAs). Thus, we studied EAAT-expression and its impact to PC-activity in NPC1^−/–^mice. Western blot revealed reduced EAAT1, EAAT2, EAAT4, and βIII-spectrin levels in NPC1^−/–^mice. EAATs play a crucial role in synaptic transmission, thus we were interested in the impact of the reduced EAAT-expression on the function of PCs. Patch-clamp recordings of PCs showed no differences in the firing patterns of NPC1^+/+^and NPC1^−/–^mice using a low internal chloride concentration. Because EAAT4 also comprises a chloride permeable ion pore, we perturbed the chloride homeostasis using a high internal chloride concentration. We observed differences in the firing patterns of NPC1^+/+^and NPC1^−/–^mice, suggesting an impact of the altered EAAT4-expression. Additionally, the EAAT-antagonist DL-TBOA acts differently in NPC1^+/+^and NPC1^−/–^mice. Our data support the line of evidence that an altered EAAT-expression/function is involved in neurodegeneration of PCs observed in SCAs. Thus, we suggest that similar pathogenic mechanisms contribute the loss of PCs in NPC1.

## Introduction

Niemann Pick Type C1 (NPC1) is a recessive hereditary lysosomal storage disease with an incidence of 1:100,000 newborns^1^. The mutations in the NPC1 gene which cause the disease reduce the function of the lysosomal/late endolysosomal cholesterol transporter NPC1. This leads to intracellular accumulation of cholesterol as well as other lipids such as gangliosides and sphingosines^1^. Patients present neurological symptoms such as cerebellar ataxia, caused by degeneration and loss of Purkinje cells (PCs)^1^. Purkinje cells play a major role in movement coordination and are the sole output of the cerebellar cortex. Loss of these cells or a disturbance in their activity lead to ataxia, a pathological hallmark, not only of NPC1^1^, but hereditary spinocerebellar ataxias (SCAs)^2^. In the pathogenesis of SCAs βIII spectrin, a cytoskeletal protein, is discussed to be a key player in a common pathogenic pathway^3^. βIII spectrin plays a crucial role in the maintenance of dendritic structures of PCs and the trafficking and localization of ion channels and EAATs, acting as glutamate transporters^3^, which in turn are involved in the modulation of the intrinsic activity of cerebellar PCs^4^. In regards of NPC1 a reduced expression of EAAT3 in the hippocampus is described for NPC1^−/−^ mice^5^, and EAAT1 in Bergmann glia of NPC1^nmf164^ mice^6^, but no data are available for PCs of the cerebellum of NPC1 deficient mice. Thus, we examined the expression of EAAT1, EAAT2, and EAAT4 in the cerebella of NPC1^−/−^ mice and studied the impact on Purkinje cell function.

## Results

### Cerebellar expression of glutamate transporters is reduced in NPC1^−/−^

A hampered function and/or loss of cerebellar glutamate transporters play a crucial role in the pathology of e.g. spinocerebellar ataxias^3,7^, suggesting a contribution to the ataxia observed in NPC1. Therefore, we analyzed the cerebellar expression levels of the Purkinje cell (PC) specific excitatory amino acid transporter 4 (EAAT4), the on glia cell located EAAT1 and EAAT2, as well as cytoskeletal protein βIII spectrin, acting as an anchor for EAAT4^8^. Semi-quantitative western blot was used to analyze whole cerebella from p55 NPC1^+/+^ and NPC1^−/−^ mice.

**Figure 1:**
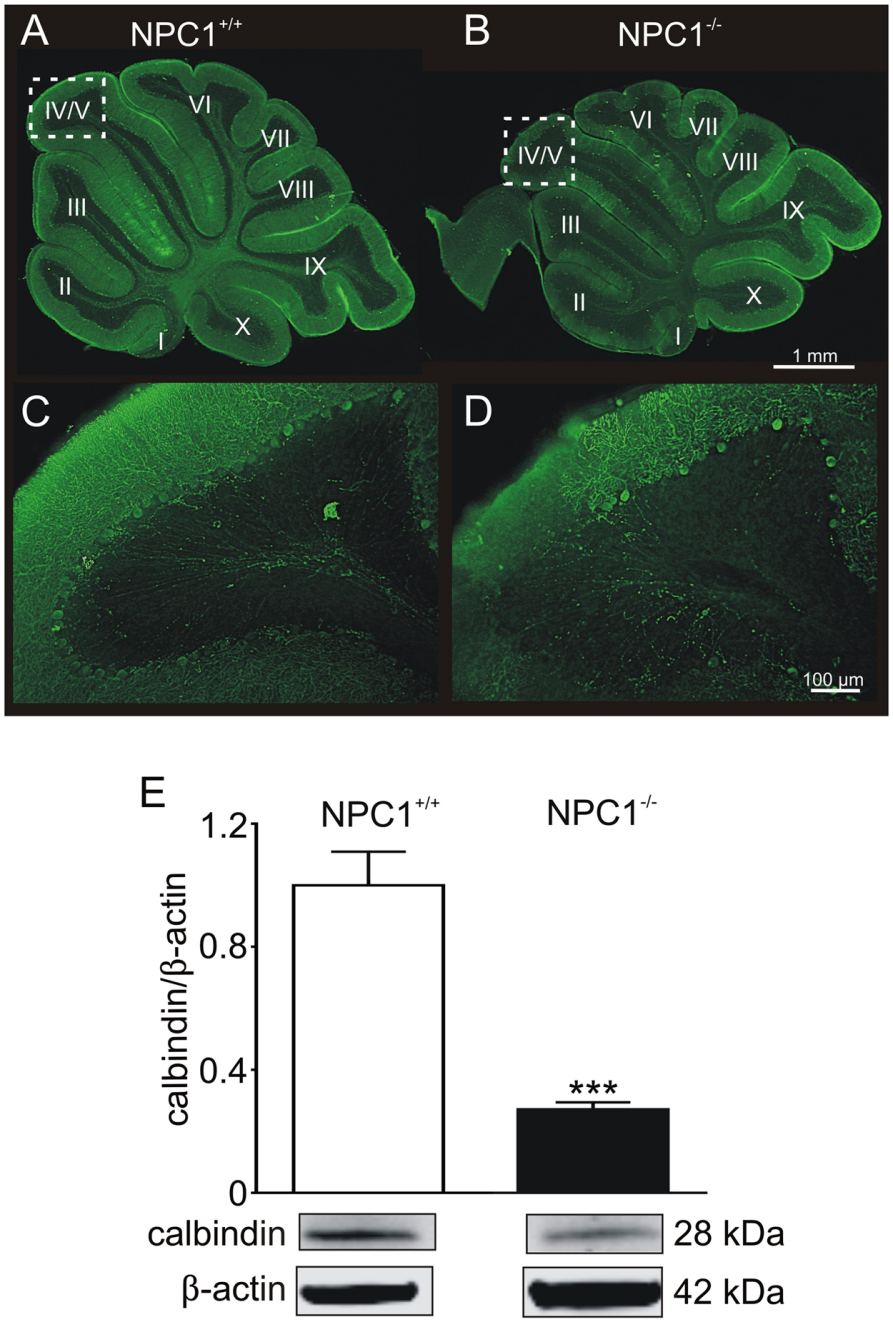
Purkinje cell degeneration in p55 NPC1^−/−^ mice NPC1^+/+^ mice. (**A**) show an even distribution of calbindin positive PCs in contrast to NPC1^−/−^ mice (**B**). A higher magnification of lobe IV/V of NPC1^+/+^ (**C**) and NPC1^−/−^ mice (**D**) demonstrates the loss of Purkinje cell somata in NPC1^−/−^ mice. (**E**) Western blot analysis confirmed a reduced number of PCs by a significantly decreased protein level of calbindin (NPC1^+/+^: N = 8, n = 25; NPC1^−/−^: N = 8, n = 23). Western blot bands display corresponding examples of the same gel. ***p < 0.001, data shown as mean ± sem. Student´s unpaired t-test was used to determine significance.

**Figure 2:**
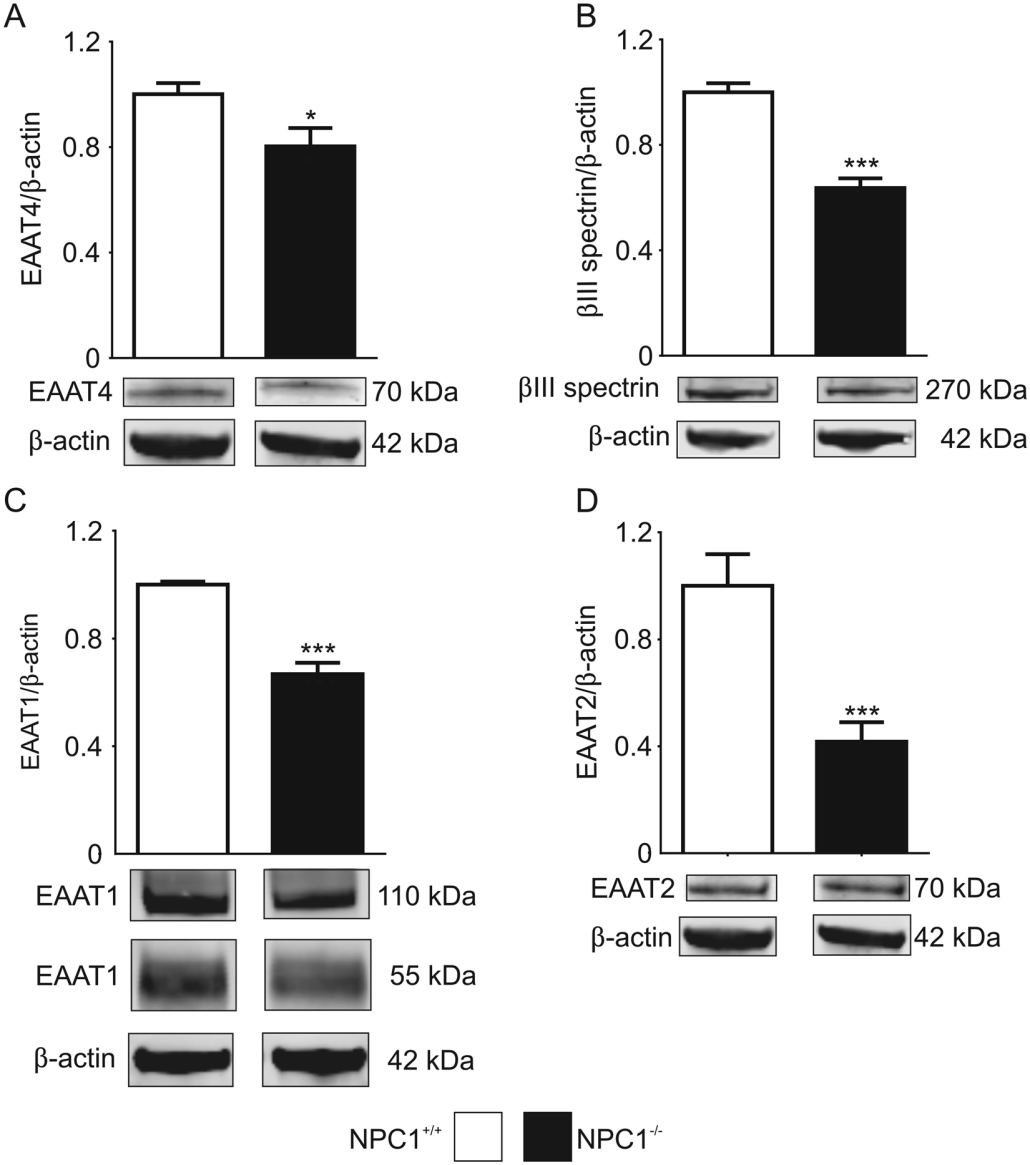
EAAT expression in cerebella of NPC1^+/+^ and NPC1^−/−^ mice. (**A**) Expression of PC specific EAAT4 (NPC1^+/+^: N = 8, n = 25; NPC1^−/−^: N = 8, n = 23) as well as (**B**) the EAAT4 stabilizing protein βIII spectrin (NPC1^+/+^: N = 8, n = 25; NPC1^−/−^: N = 8, n = 24) were significantly reduced in NPC1^−/−^ mice. (**C**) Expression of on glia cell located EAAT1 (NPC1^+/+^: N = 5, n = 10; NPC1^−/−^: N = 5, n = 8) and (**D**) EAAT2 (NPC1^+/+^: N = 8, n = 16; NPC1^−/−^: N = 8, n = 13) was also significantly reduced in NPC1^−/−^ mice. Bar graph of EAAT1 shows the sum of EAAT1 110 kDA and 55 kDa band. Western blot bands display corresponding examples of the same gel. *p < 0.05, ***p < 0.001, data shown as mean ± sem. Student´s unpaired t-test was used to determine significance.

In accordance with recent studies^9,10^, in NPC1^−/−^ mice we found a graduated loss of calbindin positive PCs from the anterior lobes to the posterior lobes, wherein lobe X appears to be unaffected (Fig. 1A), which is in accordance with previous studies^11,12^. In pictures with a higher magnification of lobe IV/V (Fig. 1C,D, areas indicated by dotted rectangle in A and B) one can observe a reduced number of Purkinje cells in NPC1^−/−^ mice. Determination of protein level revealed a significantly reduced amount of calbindin (Fig. 1E). Regarding the expression of the EAATs, we observed a significantly reduced amount of EAAT4 and the EAAT4 stabilizing cytoskeletal protein βIII spectrin (Fig. 2A,B). The protein level of the glial EAAT1 and EAAT2 was also significantly reduced (Fig. 2C,D). EAATs, acting as glutamate transporters, are not only crucial for the modulation of synaptic transmission, e.g. by the uptake of glutamate from the synaptic cleft, but can influence the activity of the cells due to their dual action, comprising the function of a transporter as well the function of an Cl^−^ permeable ion channel such as EAAT4^13^. As we observed significantly reduced expression of EAATs in NPC1^−/−^ mice, we were interested in the impact on the function of Purkinje cells.

### Purkinje cell activity pattern depends on internal chloride concentration

**Figure 3:**
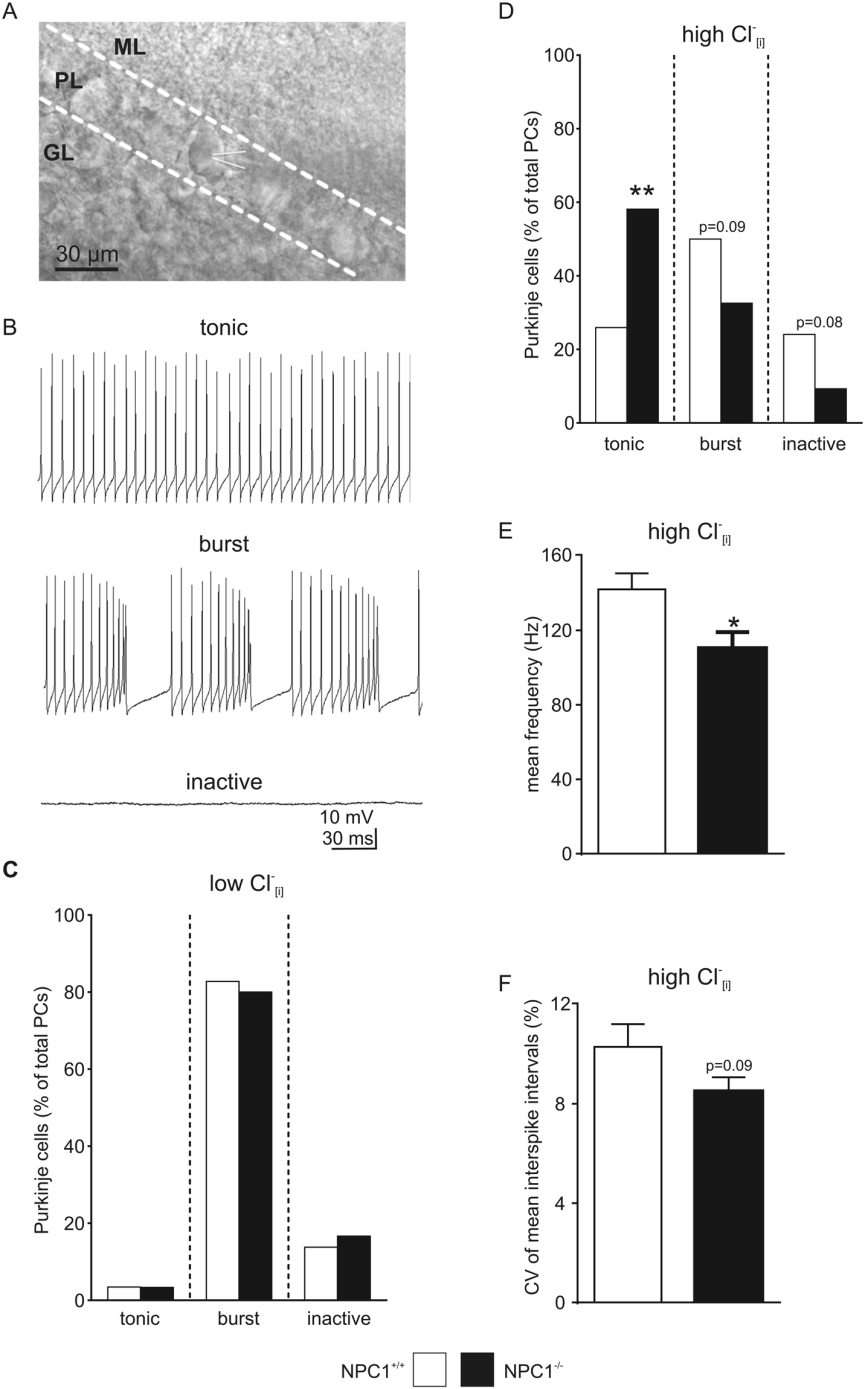
Chloride-dependent alterations of Purkinje cell activity pattern. (**A**) Optical identified PCs in lobes III-V were used for whole-cell patch clamp recordings. Recording electrode attached to PC is depicted by white lines. (**B**) Using the current clamp mode, three different activity patterns of PCs were found: tonic firing, burst firing and inactive. (**C**) No differences in pattern distribution between NPC1^+/+^ and NPC1^−/−^ mice were observed using a low Cl^-^_[i]_ (NPC1^+/+^: N = 11, n = 29; NPC1^−/−^: N = 8, n = 30). (**D**) Significantly more tonic firing PCs were found in NPC1^−/−^ mice using a high Cl^−^_[i]_, whereas more PCs in NPC1^+/+^ mice showed burst activity or were inactive (NPC1^+/+^: N = 19, n = 52; NPC1^−/−^: N = 15, n = 43). (**E**) The tonic firing PCs of NPC1^−/−^ mice showed a significantly reduced action potential frequency and (**F**) a higher spike regularity using a high Cl^-^_[i]_ (NPC1^+/+^: N = 12, n = 14; NPC1^−/−^: N = 11, n = 25). *p < 0.05, **p < 0.01, data shown as mean ± sem. ML = molecular layer, PL = Purkinje layer, GL = granular layer. Barnard’s 2 × 2 test was used to determine significance for data shown in C and D. Student´s unpaired t-test was used to determine significance for data shown in E and F.

To check the functional impact of EAAT downregulation, we recorded the action potential firing pattern of PCs of mice between p45 and p55. Firing pattern of optical identified PCs in lobe III-V (Fig. 3A) were recorded by means of whole-cell patch clamp recordings using the current-clamp mode. Recordings revealed different activity patterns, and we defined three groups of PCs: tonic firing, burst firing, and inactive cells (Fig. 3B). Using a low internal chloride concentration (Cl^−^_[i]_) 80% of the PCs (NPC1^+/+^: 24/29, NPC1^−/−^: 24/30) generated action potentials in bursts, independent of the genotype (Fig. 3C). Only a few cells showed a tonic pattern (NPC1^+/+^: 1/29, NPC1^−/−^: 1/30) or were inactive (NPC1^+/+^: 4/29, NPC1^−/−^: 5/30). Because we found a significantly reduced expression of EAAT4, and due to the intrinsic Cl^−^ conductibility of the transporter, we decided to perturb the Cl^−^ homeostasis using a high Cl^−^_[i]_. This revealed a shift from burst firing pattern to tonic firing pattern (Fig. 3D). Moreover, the activity pattern distribution was different between NPC1^+/+^ and NPC1^−/−^ mice, wherein significantly more tonic firing cells were observed in NPC1^−/−^ mice (NPC1^+/+^: 14/52, NPC1^−/−^: 25/43), whereas less cells were generating bursts (NPC1^+/+^: 27/52, NPC1^−/−^: 14/43) or were inactive (NPC1^+/+^: 13/52, NPC1^−/−^: 4/43). The analysis of the tonic firing pattern revealed significantly reduced AP frequencies in PCs of NPC1^−/−^ mice (Fig. 3E) and a lower CV of mean interspike intervals (Fig. 3F). The latter suggests that PCs of NPC1^−/−^ mice generate action potentials more regularly than the PCs of NPC1^+/+^ mice.

**Figure 4:**
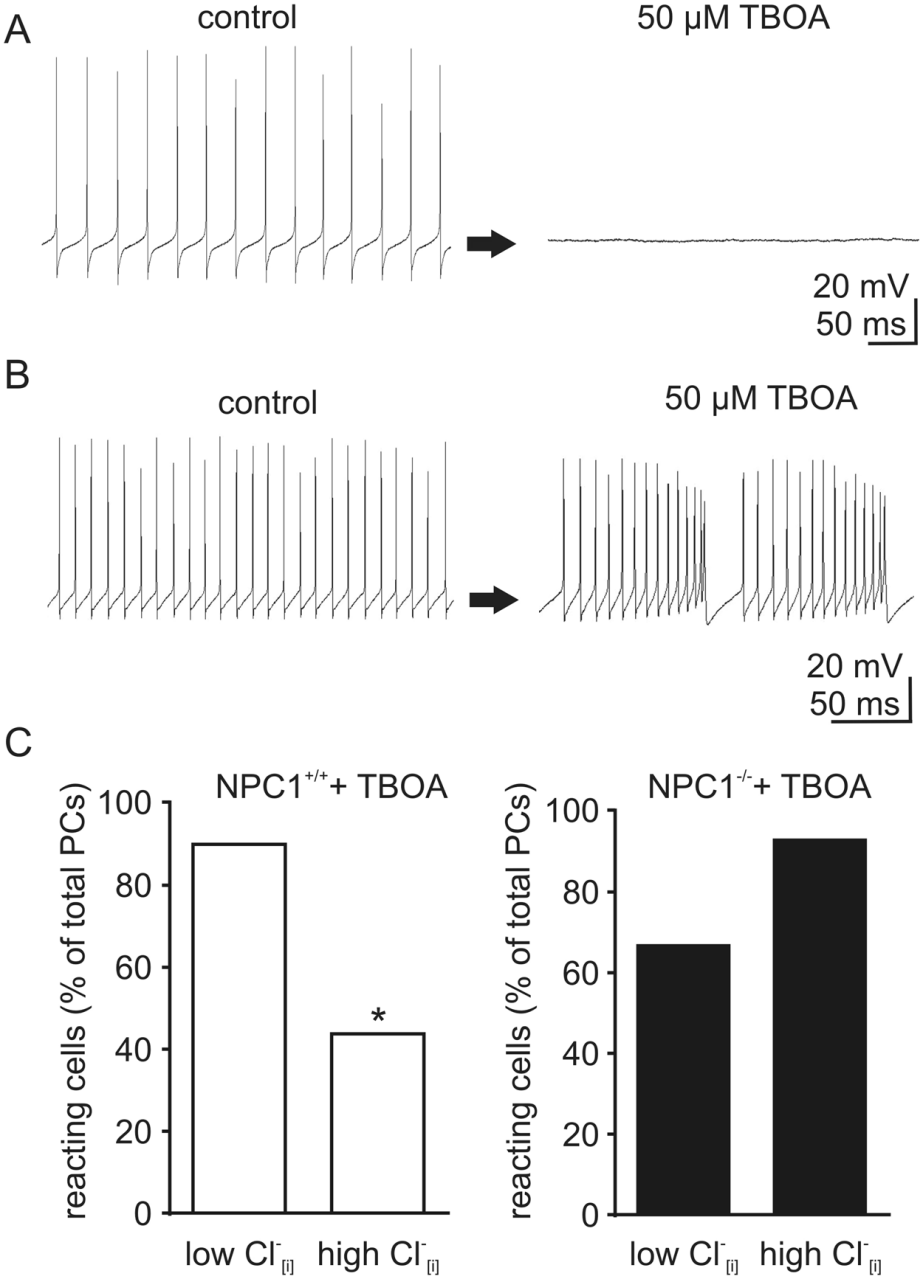
Chloride-dependent effect of TBOA on Purkinje cell activity pattern. (**A**) Example traces of TBOA induced change of activity pattern from tonic firing to inactive and (**B**) tonic to burst firing. (**C**) Applying TBOA, a significantly reduced number of PCs in NPC1^+/+^ mice changed their activity pattern using different Cl^−^_[i]_ (Low Cl^−^_[i]_: N = 3, n = 10; high Cl^−^_[i]_: N = 7, n = 16)_._ No such effect was observed in NPC1^−/−^ mice (Low Cl^−^_[i]_: N = 5, n = 12; high Cl^−^_[i]_: N = 5, n = 14). *p < 0.05. Barnard’s 2 × 2 test was used to determine significance for data shown in C.

To further address the impact of reduced EAAT4 expression on the activity pattern, we applied 50 µM of the EAAT inhibitor DL-TBOA (TBOA). We observed various effects of TBOA on the activity pattern, e.g. a shift from tonic firing to inactive (Fig. 4A) or an alteration of frequency, length of bursts, or interburst intervals (Fig. 4B). Independent of the kind of alteration, we determined the percentage of cells affected by TBOA using once again a low and a high Cl^−^_[i]_ to perturb the Cl^−^ homeostasis. In 90% (9/10) of the NPC1^+/+^ PCs, TBOA induced changes in the activity pattern with a low Cl^−^_[i]_, while the number of cells reacting was significantly lower (44%, 7/16), with a high Cl^−^_[i]_ (Fig. 4C). In contrast, only 67% (8/12) of the NPC1^−/−^ Purkinje cells were affected by TBOA using a low Cl^−^_[i]_, but when using a high Cl^−^_[i]_ 93% (13/14) of the PCs reacted to TBOA, although the difference was not significant (Fig. 4D). In sum, we noticed a reduced cerebellar expression of glial glutamate transporters EAAT1, EAAT2, the PC specific EAAT4, and βIII spectrin in NPC1^−/−^ mice. Furthermore, we found Cl^−^ dependent alterations in the activity pattern distribution of Purkinje cells as well as in the number of Purkinje cells showing a TBOA-induced activity pattern switch. These data suggest that the modulation of the activity of cerebellar PCs by EAATs is altered in NPC1^−/−^ mice.

## Discussion

Purkinje cells are the sole output of the cerebellar cortex. Loss of these cells or a disturbance in their activity lead to ataxia, a pathological hallmark of NPC1^1^ or hereditary spinocerebellar ataxias (SCAs)^2^. In the pathogenesis of SCAs βIII spectrin, a cytoskeletal linker protein, is discussed to be a key player in a common pathogenic pathway^3^. βIII spectrin plays a crucial role in the maintenance of dendritic structures of PCs and the trafficking and localization of ion channels and EAATs^3^, which in turn are involved in the modulation of the intrinsic activity of cerebellar PCs^4^. Thus, we were interested in alterations of the expression of βIII spectrin and EAATs in the cerebellum of NPC1^−/−^ mice and the impact on the intrinsic activity of cerebellar PCs.

The here observed lower expression of βIII spectrin is in line with findings in different SCAs and supports the idea of a common pathogenic pathway for cerebellar ataxias^3^, wherein alterations of the cytoskeleton lead to dislocation of e.g. ion channels or transporters like EAATs. In regards of NPC1 other cytoskeletal alterations like hypophosphorylation of vimentin^14–16^ or GFAP^16^ are described, as well as changed expression of e.g. excitatory AMPA receptors^17^. Regarding EAATs, βIII spectrin stabilizes EAAT4 in the membrane of PCs and a loss of βIII spectrin affects EAAT4 expression as described for SCA5^7^. A lower EAAT4 expression is also described for the *staggerer* mice, a model system for SCA1^18,19^. Thus, one can speculate that the here observed decreased protein amount of EAAT1, EAAT2 and EAAT4 is based on the decreased amount of βIII spectrin. Another reason for the decreased EAAT amount might be due to a reduced number of granulare cells, observed in NPC1, and thus a reduced number of synapses established between parallel fibers and PCs^20,21^. However, the underlying mechanism of the reduced βIII spectrin amounts stay elusive and needs further examination. For further studies it might also be of interest to focus on EAATs located in glial cells as e.g. in SCA5, PC degeneration is proposed to be induced by a loss of EAAT1, located on Bergmann glia cells^7^. Interestingly, Bergmann glia reduce their EAAT1 expression during disease progression, without expressing the mutated βIII spectrin themselves. It was proposed that this is caused by an altered communication between PCs and Bergmann glia cells^7^. This point is especially interesting for NPC1 as the role of glial cells in NPC1 is contradictory. A neuron specific deletion of the NPC1 gene induced neurodegeneration in mice, but not a glia cell specific deletion^11^. In contrast, a knock in of the normal gene in glia cells was sufficient to extended the survival of NPC1^−/−^ mice^22^. Here, we demonstrated a reduced amount of EAAT1 and EAAT2, located in the cerebellum mainly on glial cells, and EAAT4, mainly located on PCs, suggesting a contribution of both cell types to the pathogenic mechanism. However, the impact of a *NPC1* mutation seems to be region specific, as a EAAT3 downregulation but an unaltered EAAT1 and EAAT2 expression was found in the hippocampus of NPC1^−/−^ mice^5^, in contrast to the here observed changes of EAAT1 and EAAT2.

To determine the functional impact of reduced EAAT and βIII spectrin expression, we checked the action potential generation of PCs, which is affected in several ways in different ataxias. Changes in the firing pattern distribution, e.g. in SCA2 and SCA3^23,24^, altered action potential frequency, e.g. in SCA2, SCA3, and SCA5^7,23,24^, or a higher action potential irregularity, as described in SCA2 and episodic ataxia type 2^23,24^ show the pathophysiological heterogeneity of hereditary ataxias. Unlike these studies, describing different activity pattern of PCs in wild type and mutant mice, we observed no such differences between NPC1^+/+^ and NPC1^−/−^ mice using a low Cl^−^_[i]_, reflecting the physiological situation. This is in accordance with a previous study, using a mouse model with a specific deletion of NPC1 in PCs, were also no alterations in PC activity was found^25^. But, as EAAT4, which is predominantly expressed in the cerebellum by PCs, acts not only as a glutamate transporter but also comprises a Cl^−^ permeable ion pore^13^, we used a high Cl^−^_[i]_ to perturb the Cl^−^ homeostasis of PCs. And indeed a high Cl^−^_[i]_, reversing the effect of Cl^−^ mediated currents from inhibitory to excitatory, did result in a change of firing pattern, indicating an altered chloride conductibility of the PCs. Furthermore, the different effect of EAAT inhibition, on the activity pattern between the genotypes suggests, that EAATs can contribute to the observed alterations in different ways. Firstly, considering the Cl^−^ conductibility of EAAT4, even a moderate loss of 20% of the protein in NPC1^−/−^ mice, contributes to an alteration of the overall Cl^−^ conductibility in PCs. Secondly, not only the loss of EAAT4, but also of EAAT1 and EAAT2 could affect the synaptic transmission, due to a decreased glutamate uptake and a subsequently increased glutamate level in the synaptic cleft. In turn, the activity of e.g. GABAergic interneurons, could be affected leading to an altered GABAergic synaptic transmission to PCs^26^, resulting in altered tuning of PCs activity. In this line, an inhibition of EAATs by TBOA could lead to an additional accumulation of glutamate and therefore differently impact the activity of PCs, as we observed in NPC1^+/+^ and NPC^−/−^ mice.

In sum, our results demonstrate a reduced level of glial and neuronal EAATs, as well as the anchoring protein βIII spectrin. We conclude that these alterations contributes to Cl^−^ dependent alterations of the activity pattern of PCs in NPC1^−/−^ mice. Our data are in line of evidence with an altered function and/or expression of EAATs playing a role in the neurodegenerative mechanisms leading to a loss of PCs observed in spinocerebellar ataxias. Thus, we suggest that similar pathogenic mechanisms contribute the progressive loss of Purkinje cells in NPC1.

## Methods

### Animal housing

Heterozygote mice of the BALB/c_Nctr-Npc1m1N/-J strain^27^ (Jackson Laboratories, USA), housed in accordance with German animal welfare law, were bred to obtain homozygous NPC1-deficient (NPC1^−/−^) and unaffected, wild type (NPC1^+/+^) animals. The animals had access to food and water ad libidum. A 12 h light/dark cycle was maintained and room temperature was set to 22 °C. Tail tip samples were used to determine the genotype of the animals by polymerase chain reaction.

### Preparation of cerebellar slices

Preparation of parasagittal cerebellar vermis slices was adapted from Bischofberger *et al*.^28^. Mice were decapitated and brains were removed rapidly and incubated in an ice-cold buffer containing (mM): NaCl 125, KCl 2.5, CaCl_2_xH_2_O 2, MgCl_2_x6H_2_O 1, NaHCO_3_ 26, NaH_2_PO_4_xH_2_O 1.25, glucosexH_2_O 25, pH 7.4. After separation of the cerebellum and removal of the cerebellar hemispheres, 250 µm thick slices were cut with a vibratome (Leica VT 1200 S) in the buffer and incubated for 30 minutes at 37 °C. All steps were performed with a buffer supplied with carbogen (95% O_2_, 5% CO_2_).

### Patch clamp recordings

Patch clamp recordings of optically identified Purkinje cells were performed using an EPC-10 amplifier controlled by PatchMaster software (Heka, Germany). Patch pipettes were pulled from borosilicate glass tubing (GC150F-10, Harvard Apparatus, USA) using a DMZ-Universal-Electrode-Puller (Zeitz, Germany). The intracellular solution with a low Cl^−^ concentration IC_low_ contained (mM): K-D-Gluconate 130, KCl 10, HEPES 10, EGTA 11, MgCl_2_x6H_2_O 1, CaCl_2_x2H_2_O 1. The intracellular solution with a high Cl^−^ concentration (IC_high_) contained (mM): KCl 140, HEPES 10, EGTA 11, MgCl_2_x6H_2_O 1, CaCl_2_x2H_2_O 1. The pH was adjusted to 7.2 with KOH in both solutions. The electrodes had a resistance of 5–7 MΩ when filled with IC_low_ and 3–5 MΩ with IC_high_. Recordings of PC activity were made in the whole cell configuration in the current clamp mode with a holding current of 0pA at 34 °C. To validate the impact of EAAT function on the PC activity, EAATs were inhibited in a subset of experiments using DL-TBOA (Tocris Bioscience, United Kingdom). Data were filtered at 3 kHz and digitized with 10 kHz using PatchMaster software (Heka, Germany). Mini Analysis 6.0.7 (SynaptoSoft, USA) was used to detect action potentials and determine the activity pattern of the PCs. Frequency and coefficient of variation (CV) of mean interspike intervals were analyzed in R 3.1.2 (The R Foundation for Statistical Computing, Austria) with RStudio 0.98.1091 (RStudio, Inc., USA).

### Western blot

For western blot analysis, whole cerebella were frozen in liquid nitrogen and stored at −80 °C. For protein extraction, cerebella were placed in a tissue grinder and 1 ml RIPA-lysis buffer (in mM: TRIS 20, NaCl 137, sodium deoxycholate 12, EDTA 2, 0.1% SDS, 1% Triton® X-100, 10% glycerol supplemented with cOmplete™, Mini, EDTA-free Protease Inhibitor Cocktail (Roche Diagnostics GmbH, Germany) was added. After grinding the cerebella, the samples were incubated on ice for 30 min. The samples were centrifuged at 15,000 g for 30 min at 4 °C and the pellet was discarded. Protein concentration was determined using the Pierce™ BCA Protein Assay Kit (Thermo Fisher Scientific, USA) according to the manual. Samples were boiled for 10 minutes at 95 °C in 5 × Laemmli-buffer (125 mM TRIS, 20% glycerol, 2% SDS, 5% β-mercaptoethanol, 10% bromphenol blue) and centrifuged at 17,530 g for 1 min at 4 °C. Proteins were separated using the Criterion™ Vertical Electrophoresis Cell with Criterion™ TGX Stain-Free™ Precast Gels (4–15%) (Bio-Rad Laboratories, Germany). The electrophoresis buffer contained 25 mM TRIS, 200 mM glycine, and 0.1% SDS. For Western blot the Trans-Blot® Turbo™ Transfer System with Trans-Bolt^®^ Turbo™ Transfer Pack (Midi Format, 0.2 µm Nitrocellulose) (Bio-Rad Laboratories, Germany) was used. Membranes were washed in TRIS-buffered saline (TBS), containing 20 mM TRIS and 137 mM NaCl, pH 7.5) for 5 min and then blocked with 5% skim milk powder in TBS supplemented with 0.1% Tween^®^ 20 (TBST) for 1 h. Then membranes were incubated with a primary antibody solution (3% skim milk powder in TBST) for 1 h, washed 3 × with TBST, and incubated with DyLight™ secondary antibody for 1 h. Finally, membranes were washed 3 × with TBST and 1 × with TBS and dried. The Odyssey Infrared Imaging System (LI-COR Biosciences GmbH, Germany) was used to visualize and quantify the protein signals. Antibodies used for western blot: EAAT1 (1:5000), EAAT4 (1:1000 both Proteintech Group, USA), EAAT2 (1:1000) Calbindin d28k (1:500, both Santa Cruz Biotechnology, USA), βIII spectrin (1:2000, Thermo Fisher Scientific, USA), β-actin (1:10,000, Sigma-Aldrich, Germany), GAPDH (1:10,000, Abcam, UK), Anti-Rabbit IgG (H&L), DyLight™ 680 (1:10,000), Anti-Mouse IgG (H&L), DyLight™ 800 (1:10,000, both Rockland Immunochemicals Inc., USA). Expression of β -actin was used for normalization, and Precision Plus Protein Dual Xtra Standards (Bio-Rad Laboratories GmbH, Germany) were used as a molecular weight marker.

### Immunocytochemistry

Cerebellar brain slices with a thickness of 150 µm were prepared accordingly to the protocol used for patch clamp experiments. Staining was performed based on the protocol provided by Abcam^29^. Slices were fixed with 4% paraformaldehyde (PFA) in phosphate buffered saline (PBS) overnight at 4 °C. Fixed slices were washed 3 × for 10 min with TBS-Triton (50 mM TRIS, 150 mM NaCl, pH 7.5 supplemented with 1% Triton^®^ X-100). Afterwards slices were blocked with 4% normal goat serum (NGS) in TBS-Triton for 1 h under agitation at room temperature. Primary antibodies in 1% NGS TBS-Triton were incubated overnight under agitation at 4 °C. The next day slices were washed 3 × and a secondary antibody in 1% NGS TBS-Triton was added for 1 h at room temperature. After washing, DAPI in PBS was added, incubated for 10 min at room temperature. Afterwards the slices were washed 3 × and mounted in Mowiol-DABCO (10% Mowiol^®^ 4–88, 2.5% DABCO (1,4-diazabicyclo[2.2.2]octane), 25% glycerol, 0.1 M Tris-HCl (pH 8.5). Slices were visualized using a BZ-8000K microscope (KEYENCE, Germany). Pictures of the higher magnification (Fig. 1C,D), consist of z-stacks of 18 single pictures and were merged using the Full Focus function of the Analyser Software (KEYENCE, Germany).

### Statistical analysis

All data were obtained from at least three animals of both genotypes. The number of animals is given as “N”, the number of individual experiments is given as “n”. Analysis of the data was carried out with GraphPad Prism 6 (GraphPad Software Inc., USA). and R 3.1.2 (The R Foundation for Statistical Computing, Austria). Data are given as mean ± sem. Unless otherwise stated, unpaired Student´s t-test was used to test for significance of two sets of data differing in one variable (GraphPad Prism 6, GraphPad Software Inc., USA). Barnard’s 2 × 2 test^30^ was used to compare the two variables of data of the experimental settings comparing activity pattern differences in NPC1^+/+^ and NPC1^−/−^ mice. P-values < 0.05 were considered statistically significant, with *p < 0.05, **p < 0.01, and ***p < 0.001.

### Ethics approval and consent to participate

Housing and breeding of animals, and experimental procedures were done in accordance with the German Animal Welfare Law (Deutsches Tierschutzgesetz). Approval was given by Landesamt für Landwirtschaft, Lebensmittelsicherheit und Fischerei Mecklenburg-Vorpommern (LALLF-MV), Rostock, Germany.

### Availability of data and material

The datasets used and/or analyzed in this study are available from the corresponding author on reasonable request.
